# Impact assessment of onchocerciasis through lymphatic filariasis transmission assessment surveys using Ov-16 rapid diagnostic tests in Sierra Leone

**DOI:** 10.1186/s13071-024-06198-5

**Published:** 2024-03-11

**Authors:** Ibrahim Kargbo-Labour, Mohamed S. Bah, Natalie V. S. Vinkeles Melchers, Abdulai Conteh, Victoria Redwood-Sawyerr, Wilma A. Stolk, Jusufu Paye, Mustapha Sonnie, Amy Veinoglou, Joseph B. Koroma, Mary H. Hodges, Angela M. Weaver, Yaobi Zhang

**Affiliations:** 1 National Neglected Tropical Disease Control Programme, Ministry of Health and Sanitation, Freetown, Sierra Leone; 2 Helen Keller International, Freetown, Sierra Leone; 3 Department of Public Health, Erasmus MC, University Medical Center Rotterdam, Rotterdam, The Netherlands; 4 Department of Social Sciences, Health and Society, Wageningen University and Research Centre, Wageningen, The Netherlands; 5 Helen Keller International, New York, USA; 6 FHI 360, Ghana Country Office, Accra, Ghana

**Keywords:** Onchocerciasis, Ov-16, Antibody, Prevalence, Sierra Leone

## Abstract

**Background:**

Onchocerciasis is endemic in 14 of Sierra Leone's 16 districts with high prevalence (47–88.5%) according to skin snips at baseline. After 11 rounds of mass treatment with ivermectin with good coverage, an impact assessment was conducted in 2017 to assess the progress towards eliminating onchocerciasis in the country.

**Methods:**

A cluster survey was conducted, either integrated with lymphatic filariasis (LF) transmission assessment survey (TAS) or standalone with the LF TAS sampling strategy in 12 (now 14) endemic districts. Finger prick blood samples of randomly selected children in Grades 1–4 were tested in the field using SD Bioline Onchocerciasis IgG4 rapid tests.

**Results:**

In total, 17,402 children aged 4–19 years in 177 schools were tested, and data from 17,364 children aged 4–14 years (14,230 children aged 5–9 years) were analyzed. Three hundred forty-six children were confirmed positive for Ov-16 IgG4 antibodies, a prevalence of 2.0% (95% CI 1.8–2.2%) in children aged 4–14 years with prevalence increasing with age. Prevalence in boys (2.4%; 95% CI 2.1–2.7%) was higher than in girls (1.6%; 95% CI 1.4–1.9%). There was a trend of continued reduction from baseline to 2010. Using data from children aged 5–9 years, overall prevalence was 1.7% (95% CI 1.5–1.9%). The site prevalence ranged from 0 to 33.3% (median prevalence = 0.0%): < 2% in 127 schools, 2 to < 5% in 34 schools and ≥ 5% in 16 schools. There was a significant difference in average prevalence between districts. Using spatial analysis, the Ov-16 IgG4 antibody prevalence was predicted to be < 2% in coastal areas and in large parts of Koinadugu, Bombali and Tonkolili Districts, while high prevalence (> 5%) was predicted in some focal areas, centered in Karene, Kailahun and Moyamba/Tonkolili.

**Conclusions:**

Low Ov-16 IgG4 antibody prevalence was shown in most areas across Sierra Leone. In particular, low seroprevalence in children aged 5–9 years suggests that the infection was reduced to a low level after 11 rounds of treatment intervention. Sierra Leone has made major progress towards elimination of onchocerciasis. However, attention must be paid to those high prevalence focal areas.

**Graphical Abstract:**

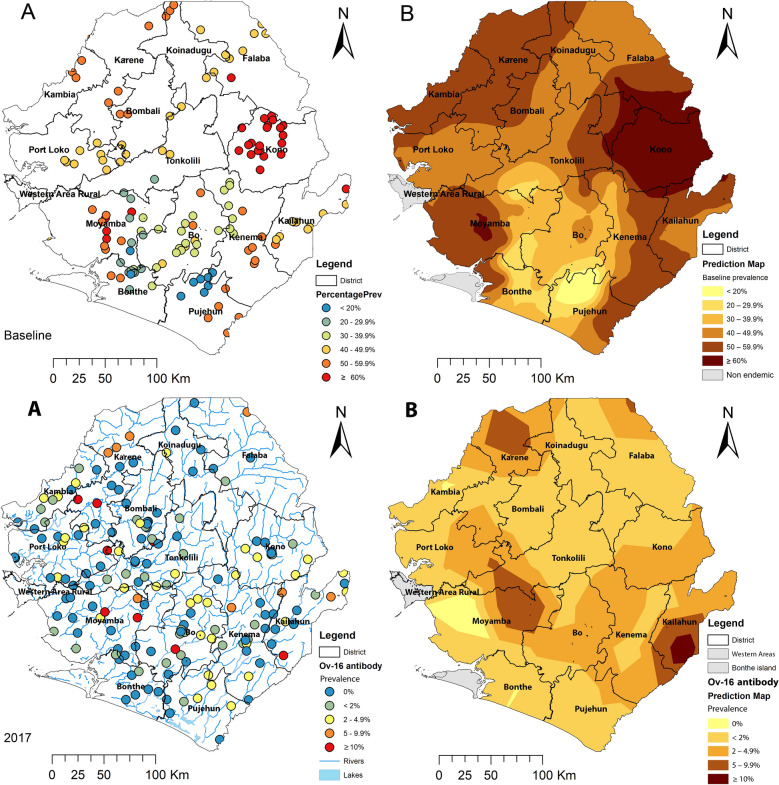

## Background

Onchocerciasis, also known as river blindness, is the world’s second leading infectious cause of blindness [1]. Human disease is caused by infection with the parasite, *Onchocerca volvulus*, and is transmitted by *Simulium* black flies. Mature adult parasites live in nodules in the soft subcutaneous tissues for approximately 10–15 years. Microfilariae (mf) produced by inseminated mature female worms migrate through the human body and have a life span of 12–15 months. Onchocerciasis pathology is mainly the result of the inflammation caused by the mf that die in different locations in the body. The skin inflammation causes, among others, itching and reactive skin disease and, in the long term, skin discoloration, atrophy and hanging groin [2]. Inflammation in the eye causes lesions on the cornea, which is reversible initially, but can progress to permanent clouding of the cornea if not treated, leading to blindness. Globally, onchocerciasis is endemic in Africa, the Americas and Yemen. It was estimated in 2021 that at least 244 million people required preventive chemotherapy, with > 99% of infected people living in 25 African countries [3]. In East Africa alone, it was estimated that by 2020 14.1 million people were mf positive out of which 8.6 million people manifested skin disease and 552,000 experienced vision loss. The total disability-adjusted life years (DALYs) lost because of onchocerciasis were estimated to be 1.4 million [4].

The Onchocerciasis Control Program (OCP) in West Africa started in 1975 to control onchocerciasis as a disease of public health importance and an obstacle to socioeconomic development in West Africa [5]. The OCP conducted endemicity mapping focusing on black fly breeding sites and conducted vector control (by larviciding) across all endemic river basins, later coupled with large-scale ivermectin distribution in high-risk communities. By its closure in 2002, the OCP supported 11 West African countries and achieved the program objectives in all 11 countries except in Sierra Leone [6]. The African Program for Onchocerciasis Control (APOC) was launched in 1995 to support 20 other African countries to control human onchocerciasis as a public health problem [7]. Communities were mapped using the rapid epidemiological mapping of onchocerciasis (REMO), and high-risk areas (with nodule prevalence ≥ 20%) were delineated for community-directed treatment with ivermectin (CDTI) [8]. Five ex-OCP countries including Sierra Leone were included from 2003 in APOC’s Special Intervention Zone (SIZ) to continue CDTI [9]. Under both OCP and APOC, epidemiological evaluations were periodically conducted at sentinel villages using skin snip methods. Field evidence from Mali and Senegal suggested that it was possible to eliminate onchocerciasis by ivermectin treatment [10, 11]. The World Health Organization (WHO) therefore transitioned the global objective from controlling onchocerciasis as a public health problem to eliminating the transmission of onchocerciasis through mass drug administration (MDA) with ivermectin [12, 13].

**Fig. 1:**
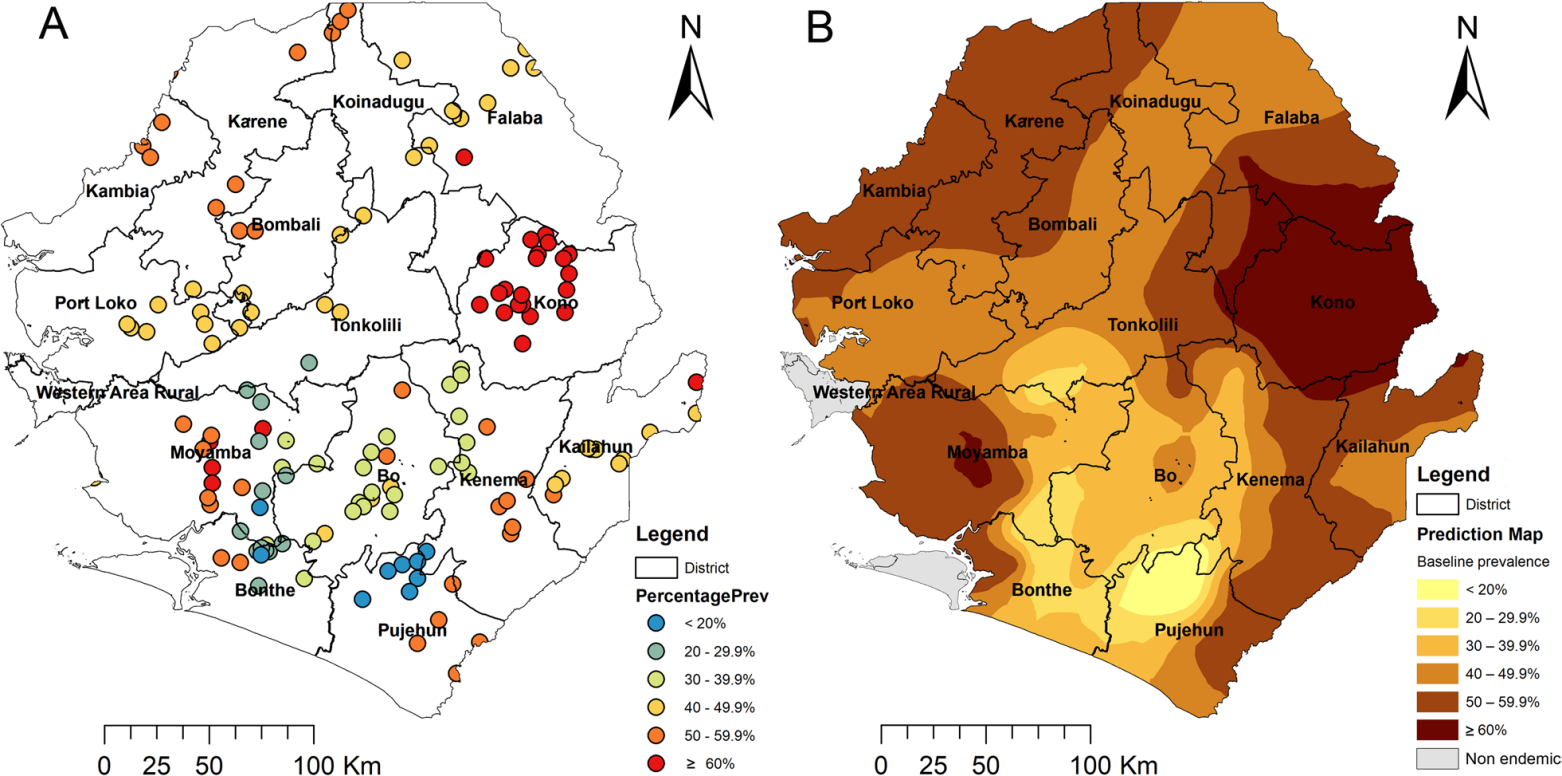
Distribution and point mf prevalence (**A**) and spatially smoothed contour maps of predicted onchocerciasis prevalence (**B**) at baseline in Sierra Leone. The mf prevalence used was from surveys conducted pre-treatment intervention in 142 sentinel villages during 1988 and 2004 in the population aged ≥ 1 year, published previously [14]. The map shows the 16 new district boundaries after redistricting: Koinadugu = Falaba + Koinadugu and Bombali + Port Loko = Bombali + Karene + Port Loko

Onchocerciasis is endemic in Sierra Leone in 14 out of 16 districts across the country. Western Area Urban and Western Area Rural in the capital, Freetown, as well as Bonthe Island, part of Bonthe District, are non-endemic [14], as shown in Fig. 1. The country was included in the OCP through the western extension (1988–2002) [15], and the whole country was designated as a SIZ under the APOC (2003–2007) [9]. Parasitological surveys by skin snip conducted across the country in 271 villages in all the major river basins before the OCP intervention showed that 88% of the villages are meso- (mf prevalence 40–59.9%) and hyper- (mf prevalence ≥ 60%) endemic with mf prevalence varying between 47 and 88.5% [15].

The National Onchocerciasis Control Programme (NOCP) in Sierra Leone was established in 1989 under OCP. A small-scale vector control program was undertaken along the Rokel River in Tonkolili between 1957 and 1959 [16, 17]. In 1989, the OCP included Sierra Leone for country-wide vector control efforts along riverbeds [18]. Larviciding was suspended in 1992/1993 in the south of Sierra Leone as the non-migratory forest-type blackfly species (*Simulium leonense*) was discovered, and it was thought that the region could only be targeted by ivermectin MDA. The north of Sierra Leone underwent larviciding of the Rokel, Mongo, Kaba, Kolente and Bagbe (tributary of Sewa) River basins for 5 years from 1990 to April 1994. Due to insecurity of the region (as a result of civil war), control activities were interrupted, and attempts to resume vector control in 1997 failed and lasted only 5 weeks [19].

MDA with ivermectin was initiated in 1990/1991 in the endemic districts in the southern region of Sierra Leone and in 1993/1994 in the endemic districts of the northern region, targeting only meso- and hyper-endemic villages, mainly those on Taia, Gbamgbaia, Jong, Sewa, Kaba, Mabole and Moago River basins [20–24]. Due to the civil war, ivermectin treatment intervention was limited from 1995 to 2002 [14, 25]. CDTI resumed in 2003 under the APOC SIZ in seven districts but with poor coverage [26]. Treatment data from five districts in 2004 were deemed unreliable because of incorrect denominators [27]. The poor results due to the apparent loss of competence and resources during the civil war prompted APOC and the Ministry of Health and Sanitation to conduct strategic reorganization of CDTI and appoint a new national program manager [28]. Treatment coverage improved from 2005 onwards, but no MDA was provided in 2014 because of the Ebola virus disease (EVD) outbreak that raged in the country. In 2007, the NOCP became the National Neglected Tropical Disease Program (NNTDP), and onchocerciasis treatment was integrated with albendazole and ivermectin MDA (as CDTI +) for lymphatic filariasis (LF) elimination in the six co-endemic districts (bordering either Guinea or Liberia) with fairly good coverage, and good treatment coverage was also achieved in the six other onchocerciasis-endemic districts by ivermectin treatment for onchocerciasis only [14, 25]. Integrated onchocerciasis/LF MDA delivered by community drug distributors was scaled up to cover all 12 onchocerciasis-endemic districts from 2008 onwards [14, 29].

WHO/APOC recommended conducting regular epidemiological evaluations to assess mf prevalence in high-risk sentinel communities near the river and the blackfly breeding sites where baseline prevalence was highest using parasitological test skin snips until a new diagnostic became available [12]. In 2010–2011, an impact assessment was conducted after five rounds of MDA at sentinel sites across the country using the skin snip method. The results showed that mf prevalence had been reduced from 53.1% at baseline to 21.1% on average, a significant reduction of 60% [14]. Detection of mf through skin snip microscopy had been the gold standard for assessing onchocerciasis. However, this invasive and painful process had met increasing resistance from communities, and it was insensitive following long-term mass treatment intervention when mf prevalence became low [30]. WHO recommends using the Ov-16 serology test for epidemiological assessment to demonstrate the interruption of transmission of *O. volvulus* in a human population testing children < 10 years old [31]. The Ov-16 rapid diagnostic test (Ov-16 RDT) was developed with sufficient sensitivity and specificity using serum samples [32, 33] and can be easily performed using finger-prick blood samples in the field [34, 35]. This rapid test detects the IgG4 antibodies to the *O. volvulus* antigen Ov-16, suggesting exposure to the *O. volvulus* parasite. The presence of such antibodies in children < 10 years of age represents active infection in this age group of children [36] and reflects the recent levels of transmission in the communities.

After another 7 years and six rounds of mass treatment (as mentioned, missing treatment in 2014 because of the EVD outbreak), we performed an onchocerciasis impact assessment in March–July 2017 using the commercially available SD Bioline Onchocerciasis IgG4 rapid test. The survey was either integrated with LF transmission assessment survey (TAS) or standalone using the LF TAS sampling strategy. The objectives were to assess the impact of the MDA intervention and to test the feasibility of integrating the onchocerciasis survey with the LF TAS. Here, we present the results of the Ov-16 assessment among children in 12 onchocerciasis-endemic districts of Sierra Leone and discuss the feasibility and limitations of the survey.

## Methods

### Treatment

Before 2000, strategies for ivermectin treatment included (1) treatment by national mobile teams on a large scale, (2) community-based treatment supported by governmental and non-governmental organizations and (3) passive treatment by local health institutions [15, 24]. Mass treatment of onchocerciasis was through CDTI from 2003 to 2007/2008 or through integrated LF/onchocerciasis MDA from 2007/2008 onward as described above and previously [14, 37]. Trained mobile teams or community drug distributors (CDDs) used dose poles to distribute ivermectin, and since 2007 ivermectin plus albendazole, to the eligible population aged ≥ 5 years within a specified period. The CDDs were members of communities that were literate and selected by their communities. Treatment was recorded using community registers or tally sheets. Before each MDA, CDDs conducted a pre-MDA census to update the community registers. After MDA, treatment numbers were summarized and submitted to health workers at the primary health units (PHU) who in turn reported the summarized treatment data to the district health management teams. The treatment data in each district were then reported to the national program.

### Impact assessment survey areas

The survey was conducted in all 12 (now 14 because of redistricting) onchocerciasis-endemic districts in Sierra Leone. All these districts were co-endemic with LF [14, 38, 39]. Eight districts qualified to conduct the first transmission assessment survey (TAS-1) for LF after five effective rounds of MDA, and therefore the LF TAS-1 was conducted in these eight districts in March 2017 using Alere Filariasis Test Strips (FTS) as described elsewhere [37]. Eight districts were grouped into four evaluation units (Bo and Pujehun, Bonthe and Moyamba, Kono and Tonkolili, Port Loko and Kambia); the TAS-1 surveys and results have been described previously [37]. Taking advantage of the opportunity, the onchocerciasis impact assessment was added and integrated into the LF TAS using Ov-16 RDT (SD Bioline, Yongin, Korea) in these eight districts. For the onchocerciasis impact assessment in the other four districts, the same LF TAS sampling strategy was used, and the four districts were grouped into two evaluation units (Bombali and Koinadugu, Kailahun and Kenema) and surveyed in July 2017.

### Survey teams and training

There were eight survey teams and four supervisors for LF TAS-1 and onchocerciasis assessment in eight districts and for the standalone onchocerciasis assessment in four districts. One team comprised a team leader, two technicians and a support staff. In addition, one community health worker was recruited locally per evaluation unit to help the survey teams navigate the communities and assist with transporting of materials from one village to another, especially in the villages that were not accessible by vehicles. Head teachers or representatives also supported the survey teams during the field work. Before the start of the survey, the training of technicians/team members and supervisors was conducted: 2 days for laboratory training and 1 day for field-based training. The objective of the training was to ensure that all protocols and laboratory test procedures were understood and followed by team members and supervisors. It also covered blood sampling and diagnostic procedures using the FTS and Ov-16 RDT.

### Selection of survey sites

The Survey Sample Builder Excel tool for LF TAS [40] was used to automatically calculate and determine the appropriate survey design and sample sizes for LF TAS-1 according to the WHO recommendations [41] and to generate a list of randomized numbers for cluster selection. Accordingly, a school-based cluster survey was conducted for LF TAS-1. A cluster survey was also selected for conducting the onchocerciasis impact assessment, as such a strategy was recommended by WHO for assessing transmission of onchocerciasis [31]. In eight districts (four evaluation units [EU]) with LF TAS-1, the same clusters (schools) for LF TAS-1 were used for onchocerciasis assessment as integrated surveys. Briefly, a comprehensive list of primary schools in each EU was obtained from the Ministry of Education, Science and Technology, and all primary schools were numbered according to geographical proximity. The schools to be surveyed were selected from the list of numbered schools by matching the list of random numbers generated by the Survey Sample Builder. In the other four districts without LF TAS1 surveys, the same LF TAS strategy was used to select clusters (schools) in two EUs for consistency.

### Selection of children

Children aged 6–7 years old (Classes 1 and 2) were needed for LF TAS tests as recommended by WHO [41], and children aged 5–9 years old were also needed for onchocerciasis tests as recommended by the WHO Onchocerciasis Technical Subgroup (OTS) [42]. In practical terms in the field, in the eight integrated LF TAS districts, children in Grades 1 and 2 were selected for both LF and onchocerciasis tests and children in Grades 3 and 4 were selected for onchocerciasis tests only, while in the other four districts without LF TAS1, children in Grades 1–4 were selected for onchocerciasis tests only. The Survey Sample Builder was also used to calculate the appropriate sampling interval and random starting number to generate two numbered lists, A and B, for selecting children. At each school, children from Grades 1–4 were assembled with help from teachers and lined up by class and sex. List A or B was randomly selected by tossing a coin and was used to select children in each line.

### Diagnostic tests

Children selected were immediately taken to the test station set up in the school. Tests of children with FTS are described elsewhere [37]. For diagnosis of onchocerciasis, the Ov-16 RDT test was conducted according to the manufacturer’s instructions. Ten microliters (10 μl) of fingertip blood was collected using a calibrated micropipette provided in the test kit from each sampled child by finger pricking with a sterile disposable lancet. The blood sample was added directly to the sample well on the test cassette. Then, four drops of chase buffer was added to the buffer well on the cassette. Each test cassette was labeled with the child’s identifier number and the start time. Test results were read after 20 min of incubation, and the results were recorded on the cassette and survey form. Due to logistical constraints of the large survey in the field, the second reading of test results at 24 h was not done. Instead, for any positive tests or invalid tests, a second confirmatory test was immediately conducted using new finger-prick blood samples of the positive children. Children were considered positive when the positivity was confirmed by the second test.

### Data collection and analysis

Treatment coverage before 2002 and treatment numbers for 2003 and 2004 were obtained from WHO/OCP/APOC reports through the WHO Institutional Repository for Information Sharing (iris) [43]. Treatment coverage was calculated using the total population censused by the mobile teams prior to treatment in the targeted villages as denominators. From 2005 onward, the annual reported treatment numbers received by the NOCP/NNTDP were used to calculate the annual treatment coverage in each district using the total at-risk population as the denominators. The total population used was the total number of people registered during the pre-MDA CDD census in rural villages and the projected population according to the national census 2004 for earlier years [44] and 2015 for later years [45] for district towns. Individual data for the impact assessment were collected on paper forms first and then entered into Microsoft Excel. The dataset was imported into the open source statistical software PSPP for analysis [46]. Chi-squared test was used to compare the differences in prevalence of Ov-16 antibody between districts, age groups and sexes. Individual data on *O. volvulus* infection for the same age group (4–14 years) at baseline and in 2010 were extracted from the data previously published [14] and analyzed for comparison. The geographical location and prevalence threshold of each survey site was plotted with the global positioning system coordinates collected for each school. Spatial analysis of the baseline mf prevalence or the Ov-16 antibody prevalence was conducted using the kriging methods in the Geostatistical Analyst Extension of the ArcGIS version 10.8.2 (ESRI, Redlands, CA, USA).

## Results

### Treatment coverage

Table 1 shows the treatment conducted and the data from 1990 to 2005. During the civil war, the national program office was looted, and the historical data were lost. Treatment data in Table 1 are from program reports available at the WHO Repository [43]. The treatment started in the southern region and then expanded to the northern region but targeted only limited river basins in meso- or hyper-endemic areas. Overall geographical coverage was very low across the country, although the program coverage was good in targeted areas (Table 1). During 1997–2000, mass treatment was either not done or data not available because of the civil war. This was similar between 2001 and 2004. Only selected villages in selected districts were treated each year with unsatisfactory treatment coverage. In 2003, total number of people treated significantly increased in seven districts (Bombali, Kailahun, Kambia, Koinadugu, Kono, Port Loko and Tonkolili); however, only three of these districts had good coverage (67.1–90.4%), with an overall program coverage of 34% and geographical coverage of 28.3% in seven districts. The other five districts (Bo, Bonthe, Kenema, Moyamba and Pujehun) were treated in 2004. Treatment coverage in 2004 was deemed unreliable with low therapeutic and geographical coverage by field investigation [27].

**Table 1:** Onchocerciasis treatment with ivermectin in Sierra Leone between 1990 and 2004^a^

Year	Treatment strategy	Treatment areas	No. of people censused	No. of people treated	Coverage (%)	Data sources
1990	Large-scale treatment by national mobile teams and community-based treatment	Taia, Gbamgbaia River Basins (537 villages)	44,274	31,230	70.5	[20]
1991	Large-scale treatment by national mobile teams and community-based treatment	Taia, Gbamgbaia and Jong River Basins	115,609	77,306	66.9	[21]
	Passive treatment by health institutions		–	180,456	–	
1992	Large-scale treatment by national mobile teams and community-based treatment	Taia, Gbamgbaia and Sewa River Basins	381,278	299,787	78.6	[22]
	Passive treatment by health institutions		–	37,476	–	
1993	Large-scale treatment by national mobile teams and community-based treatment	Kaba II, Mabo1e II and Moago I River Basins	77,778	57,321	73.7	[23, 54]
1994	Large-scale treatment by national mobile teams and community-based treatment	Six river basins	549,228	396,023	72.1	[24]
	Passive treatment by health institutions		–	33,596	–	
1995–1996	Large-scale treatment by national mobile teams and community-based treatment	962 villages	179,508	126,810	70.6	[55]
1996–1997	Large-scale treatment by national mobile teams and community-based treatment	609 villages	113,551	83,626	73.6	[56]
	Passive treatment by health institutions		–	10,732	–	
1997–1998	CDTI	Not done	–	–	–	[57]
	Passive treatment by health institutions		–	21,891	–	
1999–2000	Data not available					[58]
2001	CDTI	Bo, Moyamba, Pujehun, Kenema (1366 vilalges)	301,847	171,240	56.7	[59]
2002	CDTI	Bo, Moyamba, Pujehun, Bonthe, Kenema (1210 villages)	277,697	176,289	63.5	[60]
2003	CDTI	Kono, Tonkolili, Bombali, Koinadugu, Kambia, Kailahun, Port Loko (5154 villages)	2,269,451	771,590	34.0	[26]
2004	CDTI	Bo, Kenema, Pujehun, Moyamda, Bonthe (1653 villages)	n.a.^b^	573,749	n.a.^b^	

^a^Treatment numbers for 1990–2002 were taken from reports available from the WHO online database. The actual treatment numbers may be more than the numbers presented here from some years but could not be found

^b^It was suggested that wrong denominators were used for 2004 treatment data [27]; therefore, the actual number of people censused for 2004 was not available

**Fig. 2:**
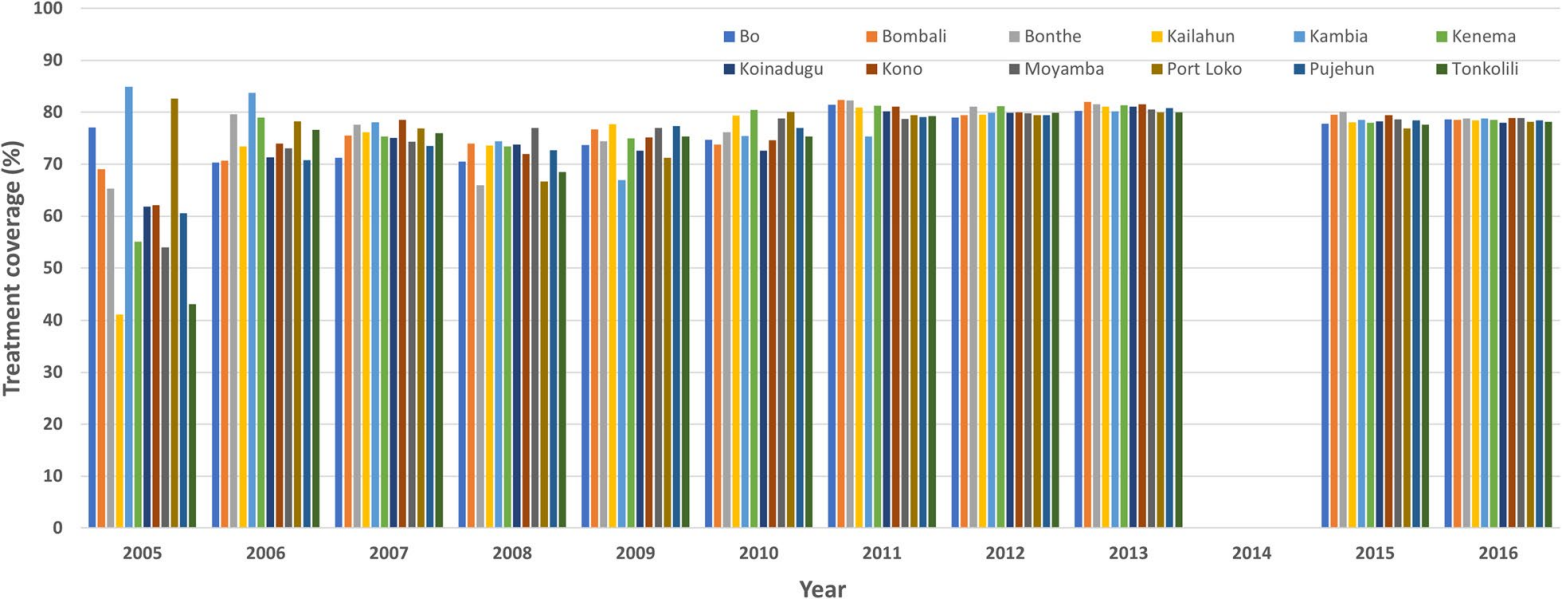
National reported treatment coverage for onchocerciasis between 2005 and 2016 in Sierra Leone

Figure 2 shows the treatment coverage from 2005 to 2016 by district. In 2005, after reorganization of CDTI and program management, treatment coverage improved, and all districts were treated. The coverage ranged from 41.1% in Kailahun to 84.9% in Kambia, with five districts achieving > 65%. From 2006 onwards, all districts achieved > 65% treatment coverage each year except 2014 when MDA was suspended because of the EVD outbreak.

### Prevalence of Ov-16 antibody in children in 2017

#### Overall prevalence in children aged 4–14 years

In total, 177 schools in 12 (now 14 because of redistricting) districts were surveyed. A total of 17,402 children were tested with Ov-16 RDT for Ov-16 IgG4 antibodies from children in Grades 1–4. Mean age of children tested was 7.8 years, ranging from 4 to 19 years old. There were 34 children aged 15–19 years old, and 4 had missing age information; these 38 children were removed from the analysis (Table 2). As a result, 17,364 children aged 4–14 years (8735 girls and 8629 boys) were valid entries and included in this analysis. There were 367 children who tested positive by the first Ov-16 RDT test, of which 346 children were confirmed positive by the second test. The confirmed positives by the second test were used for the analysis in this paper, and the overall prevalence was 2.0% [95% confidence interval (CI) 1.8–2.2%].

**Table 2:** Results of Ov-16 IgG4 antibody prevalence in children by age in 2017

Age	Number of children tested	Number of children positive by the first test	Number of children confirmed positive by the second test	Prevalence (%) (95% CI)^a^
4	5	0	0	(–)
5	1703	14	13	0.8 (0.5–1.3)
6	3550	42	38	1.1 (0.8–1.5)
7	3232	64	59	1.8 (1.4–2.4)
8	3161	78	72	2.3 (1.8–2.9)
9	2584	56	54	2.1 (1.6–2.7)
10	1782	56	54	3.0 (2.3–3.9)
11	586	20	20	3.4 (2.2–5.2)
12	511	21	20	3.9 (2.6–6.0)
13	174	8	8	4.6 (2.4–8.8)
14	76	8	8	10.5 (5.4–19.4)
(15–19)^b^	(34)	(1)	(1)	(–)
(–)^b^	(4)	(0)	(0)	(–)
Total	17,364	367	346	2.0 (1.8–2.2)

^a^Based on the confirmatory test results

^b^Number of children 15–19 years old; the ages of four children were missing so they were not included in the total

In 2017, 206 of 8629 boys tested positive with a prevalence of 2.4% (95% CI 2.1–2.7%) and 140 of 8735 girls tested positive with a prevalence of 1.6% (95% CI 1.4–1.9%) (*χ*^2^ = 13.68, *df* = 1, *P* < 0.001). The mf prevalence was 25.8% in boys (95% CI 23.4–28.4%) and 18.7% in girls (95% CI 16.5–21.2%) at baseline (*χ*^2^ = 16.28, *df* = 1, *P* < 0.001), while it was 5.3% in boys (95% CI 4.1–6.8%) and 4.6% in girls (95% CI 3.4–6.2%) in 2010 (*χ*^2^ = 0.47, *df* = 1, *P* = 0.528). There was a trend of continued reduction in *O. volvulus* infection in both boys and girls in the baseline, 2010 and 2017 data in children aged 4–14 years old.

**Fig. 3:**
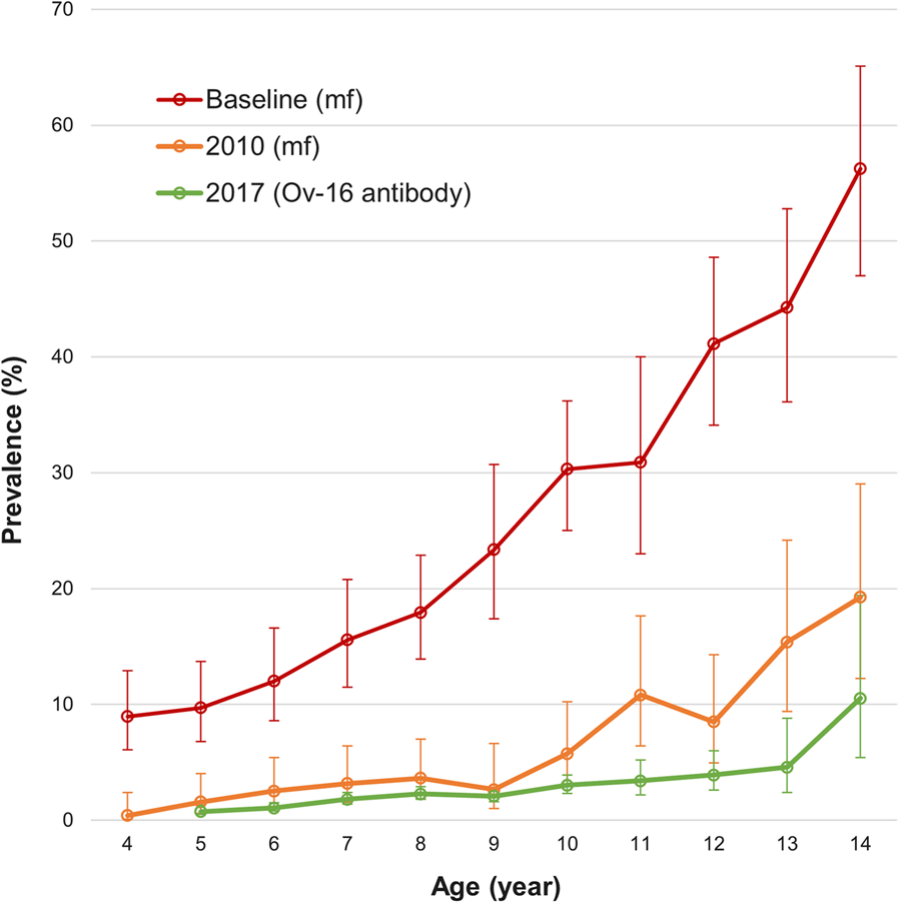
Age prevalence of *Onchocerca volvulus* infection in children aged 4–14 years at baseline, in 2010 and in 2017. Error bar represents 95% confidence interval. Only four children who were 4 years old were tested, and none were positive in 2017. Therefore, prevalence and 95% CI were not calculated for 4-year-old children

Figure 3 shows the Ov-16 IgG4 antibody age prevalence in children aged 4 to 14 years in 2017 against the mf age prevalence from the baseline and 2010 surveys. Among different ages between 4 and 14 years old, Ov-16 IgG4 antibody prevalence increased significantly with age (*χ*^2^ = 90.565, *df* = 10, *P* < 0.001). A similar trend was seen in the baseline mf prevalence (*χ*^2^ = 239.43, *df* = 10, *P* < 0.001) and 2010 mf prevalence (*χ*^2^ = 93.08, *df* = 10, *P* < 0.001). Direct statistical comparison was not made with the previous surveys as the test used in the 2017 survey was different from previous years.

#### Geographical distribution of the Ov-16 IgG4 antibody prevalence in children aged 5–9 years

**Fig. 4:**
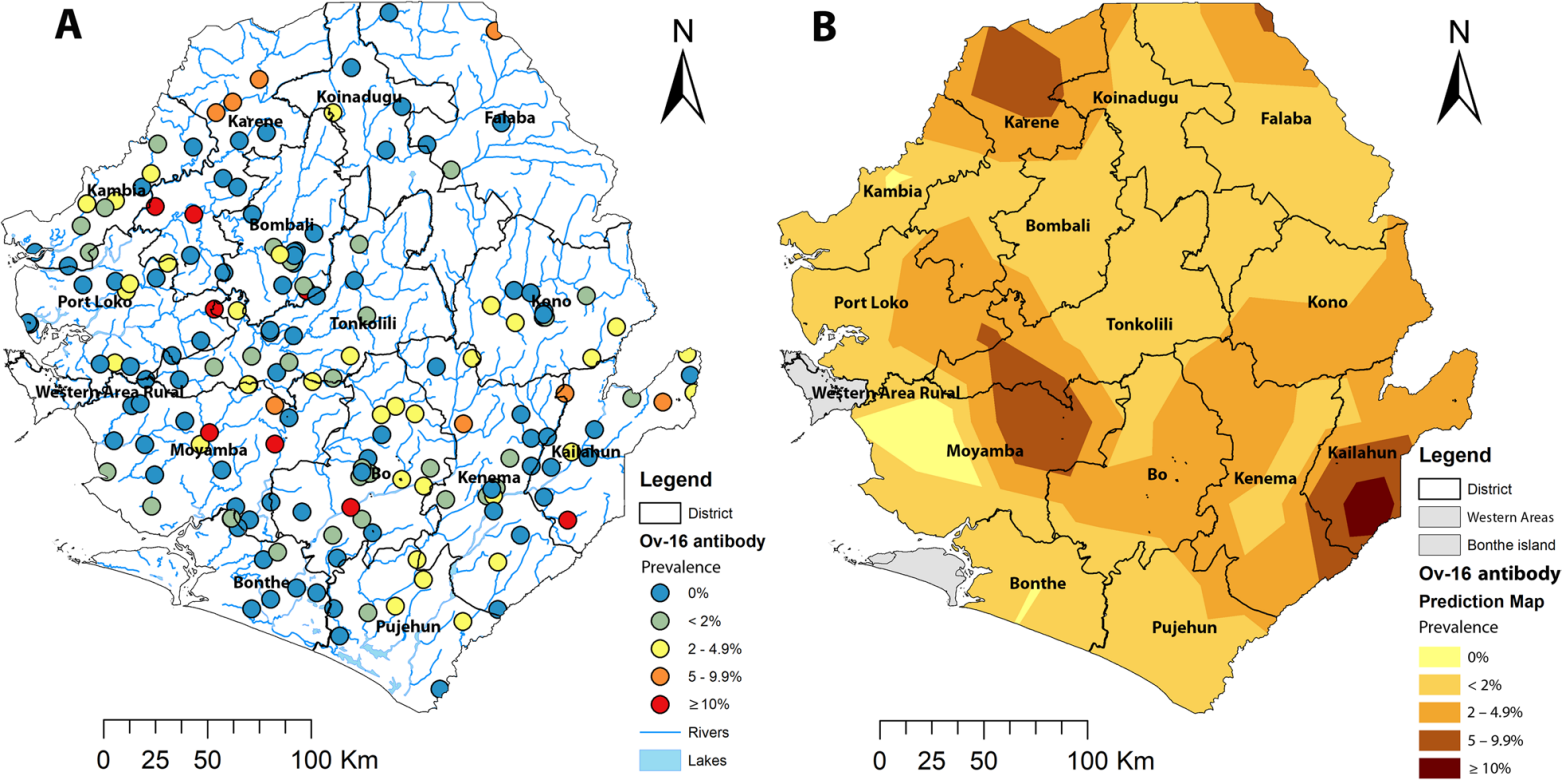
Distribution and Ov-16 IgG4 antibody point prevalence in 5–9 year olds of 177 survey sites (**A**) and spatially smoothed contour maps of predicted Ov-16 IgG4 antibody prevalence (**B**) in 2017 in Sierra Leone. The map shows the 16 new district boundaries after redistricting: Koinadugu = Falaba + Koinadugu and Bombali + Port Loko = Bombali + Karene + Port Loko

As children aged 5–9 years were recommended by WHO for impact assessment, we conducted more detailed analysis on geographical distribution in Sierra Leone using data from this age group to inform programmatic decision-making. Among children aged 5–9 years old, the mean Ov-16 IgG4 antibody prevalence was 1.7% (95% CI 1.5–1.9%). The site prevalence ranged from 0 to 33.3% (median prevalence = 0.0%) among 177 surveyed schools (Fig. 4A). One hundred twenty-seven schools (71.8% of all schools) had prevalence < 2%; an additional 34 schools (19.2%) had prevalence of 2 to < 5%, and 16 schools (9.0%) had prevalence ≥ 5%, of which 8 schools (4.5%) had prevalence > 10% (Fig. 4A).

Table 3 shows the results of each district. The mean Ov-16 IgG4 antibody prevalence in districts ranged from 0.8% in Bonthe District to 3.1% in Kailahun District. There was a significant difference in district mean prevalence between districts (*χ*^2^ = 27.68, *df* = 11, *P* = 0.004), with Bombali, Kailahun and Tonkolili > 2% and the other nine districts < 2%. Each district had one or more sites with prevalence of > 2%; particular, six districts (Bombali, Kailahun, Moyamba, Port Loko, Pujehun and Tonkolili) had sites with prevalence of > 10% (Table 3). Spatial analysis showed that the predicted Ov-16 IgG4 antibody prevalence was < 2% in coastal areas and in large parts of Koinadugu (Koinadugu + Fabala), Bombali and Tonkolili Districts, and there were some focal areas with high predicted prevalence (> 5%) centered in Karene (part of original Bombali), Kailahun and Moyamba/Tonkolili Districts (Fig. 4B).

**Table 3:** Results of Ov-16 IgG4 antibody prevalence in children aged 5–9 years by district in 2017

District	Number of clusters surveyed	Number of children tested	Number of children confirmed positive	Average prevalence (%) (95% CI)	Median prevalence (range)
Bo	16	1202	18	1.5 (1.0–2.4)	1.2 (0–4.6)
Bombali	18	1727	37	2.1 (1.6–2.9)	0.0 (0–12.8)
Bonthe	14	1079	9	0.8 (0.4–1.6)	0.0 (0–6.1)
Kailahun	11	613	19	3.1 (2.0–4.8)	2.6 (0–27.6)
Kambia	11	1061	12	1.1 (0.7–2.0)	1.0 (0–4.2)
Kenema	19	1595	18	1.1 (0.7–1.8)	0.0 (0–5.6)
Koinadugu	11	645	8	1.2 (0.6–2.4)	0.0 (0–8.3)
Kono	12	902	15	1.7 (1.0–2.7)	0.5 (0–3.8)
Moyamba	17	1235	19	1.5 (1.0–2.3)	0.0 (0–16.0)
Port Loko	19	1611	24	1.5 (1.0–2.2)	0.0 (0–19.7)
Pujehun	14	868	15	1.7 (1.1–2.8)	0.4 (0–12.5)
Tonkolili	15	1692	42	2.5 (1.8–3.3)	0.6 (0–33.3)
Total	177	14,230	236	1.7 (1.5–1.9)	0.0 (0–33.3)

^*^Data for 4–14 year olds were extracted from the baseline data (1988–2004) and the 2010 survey data, both published in Ref. [14], and data presented here are for children aged 4–14 years only

## Discussion

Impact assessment of national onchocerciasis treatment was successfully conducted in 2017 using the new diagnostic tool, Ov-16 RDT, in all 12 (now 14 due to redistricting) onchocerciasis-endemic districts in Sierra Leone. The assessment was performed as an integrated LF TAS and onchocerciasis impact assessment survey in eight endemic districts and as a standalone onchocerciasis impact assessment survey in four other endemic districts using the same LF TAS sampling strategy. Average prevalence of anti-Ov-16 IgG4 antibodies was 2.0% in children aged 4–14 years old in the country after 11 rounds of treatment intervention. It showed that boys had higher prevalence of Ov-16 antibodies than girls. This was in line with the previous observations, suggesting that boys likely have more exposure to black flies through activities closer to rivers [14]. Analysis of age prevalence of Ov-16 antibodies in children aged 4–14 years showed that the Ov-16 antibody prevalence significantly increased with age, and this may be explained by more exposure of the older children. Children aged 5–9 years had Ov-16 antibody prevalence of 1.7%, ranging from 0.8 to 2.3% in the 2017 survey across the country. As Ov-16 antibody prevalence in children < 10 years represents active infection [36], the low Ov-16 antibody prevalence in children aged 5–9 years suggests that the infection level may have been massively reduced from the baseline and further reduced since the 2010 assessment. However, direct statistical comparison between 2017 and 2010 was not made as different survey methodologies were used in both testing and sampling.

Over 70% of the schools surveyed showed an Ov-16 antibody prevalence < 2%. However, areas with > 2% prevalence were found in one or more sites in each of the surveyed districts. The areas that are endemic for onchocerciasis in Sierra Leone are considered one transmission zone [15]; therefore, it is recommended that MDA should be continued in all these districts until the next round of impact assessment in a few year’s time. Some schools showed prevalence > 5%; in particular, eight schools showed prevalence ranging from 12.2 to 33.3% (Fig. 3A). According to our geostatistical model using geospatial coordinates, areas with prevalence of ≥ 2% are predicted to be located in northern Karene (new district), a small part of the new Falaba District, a large U-shaped area spanning parts of several districts (Port Loko, Tonkolili, Moyamba, Bo, Kenema and Kono) and Kailahun. Specific focal areas in Karene, Tonkolili/Moyamba and Kailahun had prevalence > 5% in 2017 (Fig. 3B). These areas with high predicted prevalence in 2017 also had a high skin snip baseline prevalence (see Fig. 1). It is suggested that the baseline endemicity level in an area is a major factor influencing the probability of elimination of onchocerciasis even with long-term control measures [47]. Elimination of onchocerciasis transmission requires continued MDA intervention reaching all endemic communities (geographical coverage 100%) and program coverage (≥ 80%) in eligible population for at least 12–15 years, and it may take longer in areas with high pre-control endemicity levels [12, 31]. With high baseline endemicity (high prevalence and high black fly annual biting rates), limited vector control through larviciding and ivermectin treatment due to civil war interruption and poor MDA coverage post war, it may not be surprising to see that high Ov-16 antibody prevalence was found in those high baseline prevalence areas despite 12 years and 11 rounds of ivermectin treatment. However, it is noted that although Kono had the highest overall skin snip prevalence (> 50%) at baseline (Fig. 1), the 2017 assessment showed that the Ov-16 antibody prevalence was < 5% across the district (Fig. 4). Overall, great progress had been made since the baseline in the country as shown previously [14] and by data presented here.

WHO/OTS recommends testing children aged 5–9 years old for onchocerciasis impact assessment using serological tests in the first-line villages in each transmission zone: 100 children per village from 3 to 5 first-line villages [42]. This survey was conducted as a large-scale field application of the new diagnostic tool, Ov-16 RDT, before the WHO/OTS recommendation later in 2017. There was no formal recommendation at the time on sampling strategy for conducting serological impact assessment. Therefore, we used a cluster survey strategy recommended by WHO for determining the interruption of onchocerciasis transmission in this survey [31]. We took the opportunity to integrate this onchocerciasis assessment with the LF TAS-1 in eight districts and adopted the sampling strategy of LF TAS without stratifying for first- and second-line villages. To our knowledge, this was the first large-scale survey using such survey methodologies at the time. However, there may be some limitations of the study that affected the outcomes of the impact assessment, so caution is required in interpreting the current prevalence results. As mentioned above, onchocerciasis in Sierra Leone only has one transmission zone, which covers 80% of the country, and nearly 90% of all villages in Sierra Leone were hyper- or meso-endemic at baseline [15]. Therefore, the current results from a large sample provided important information on the prevalence distribution across the country, though they may have underestimated the true prevalence by not selecting only the first-line villages. The approach of LF TAS for select children (6–7 years) was made by school class (Grades 1 and 2) rather than by exact ages for practical reasons in the schools. This approach was also adopted for the onchocerciasis survey by selecting additional children from Grades 3–4 regardless of ages. This resulted in ages spanning 4 to 19 years old with many children tested outside the age range required; however, 82% of the total children tested were 5–9 years old, and data for this specific group of children were analyzed separately. Taken together, this may be another limitation when conducting onchocerciasis assessment using the LF TAS approach.

At the time of the survey, we followed the manufacturer’s instructions to conduct the tests in the field using whole blood samples but did not read the results at 24 h because of logistical constraints in the field for the large-scale survey. It was shown that reading results at 24 h increases the positivity rate [48]. By not reading at 24 h, our results may have slightly underestimated the true prevalences. The sensitivity of the Ov-16 RDT was 74.8–89.1% and the specificity was 97–98.6% using the serum samples [49, 50]. Recent studies have shown that conducting Ov-16 RDT on whole blood samples has low sensitivity but using eluates from dried blood spots in the laboratory improves performance and increases sensitivity of Ov-16 RDT [51, 52]. The whole blood samples were used in field conditions in this survey; therefore, results may have further underestimated the true prevalence in communities. This may be another limitation of this survey.

As defined by WHO, onchocerciasis elimination programs have three phases: intervention/treatment, post-treatment surveillance and post-elimination surveillance [31]. The results presented here showed that Sierra Leone was still at the intervention phase, with high prevalence in some areas and not ready to conduct pre-stop MDA assessments. WHO recommends conducting impact assessment at least every 4–5 years during this phase to assess progress [31, 42]. However, due to the limited funding resources for surveys at the time, we were not able to conduct this assessment earlier. It is important that countries have funds to conduct impact assessments as often as recommended so that programmatic issues can be identified and course correction can be made in a timely manner, or programs can quickly move on to the next phase towards their elimination goals. This survey was conducted at a time when no clear WHO guidance was available on how to conduct impact assessment using Ov-16 serological tests. It is therefore recommended that the next impact assessment should follow the WHO/OTS recommended methodology, i.e. Ov-16 serology on eluates from dried blood spots in the laboratory with samples from children aged 5–9 years old in the first-line villages [42, 51, 53].

Despite the limitations of the assessment, the results provided comprehensive understanding of the onchocerciasis distribution across the country following 11 rounds of MDA and indicated how far the onchocerciasis program had progressed in Sierra Leone. Sierra Leone has faced many challenges since the OCP, such as civil war and the EVD outbreak. However, Sierra Leone overcame these challenges and reorganized the national program and CDTI to achieve effective MDA from 2005 and has maintained good coverage since then. The results also provided evidence for areas with potential ongoing onchocerciasis transmission for the NNTDP to focus on to help Sierra Leone move closer to elimination of onchocerciasis transmission. The remaining high-prevalence areas should be the focus of the NNTDP to consider to improve MDA quality and coverage. A program review to analyze MDA coverage and CDD/community compliance at the sub-district level should be conducted to help design appropriate strategies to improve the quality of MDA. Many high prevalence areas were close to border areas as indicated in Fig. 4. Cross-border transmission has been a major challenge but becomes increasingly important when closer to the endgame of onchocerciasis elimination. The NNTDP should therefore strengthen the collaboration with national NTD programs in Guinea and Liberia to improve MDA and reduce cross-border transmission.

## Conclusions

Onchocerciasis impact assessment was successfully conducted in 12 (now 14 because of redistricting) onchocerciasis-endemic districts, integrated with the LF TAS-1 in 8 districts and using the LF TAS strategy in the other 4 districts in Sierra Leone in 2017. The results showed that the *O. volvulus* exposure in children had been reduced to a very low level after 11 rounds of successful implementation of annual mass treatment in all endemic districts, though with certain limitations on conducting onchocerciasis impact assessment integrated with LF TAS using LF TAS sampling methodology. MDA should be continued in all endemic districts as there are still some high prevalence areas scattered around the country and intensified efforts are required to retain high MDA coverage. The NNTDP should further conduct a program review to analyze MDA coverage and CDD/community compliance at sub-district level to improve the quality of MDA implementation, strengthen cross-border collaboration with Guinea and Liberia to improve MDA in border areas and to reduce cross-border transmission, and prepare for the next impact assessment following the WHO/OTS recommended impact assessment methodology.

## Data Availability

All data generated or analyzed during this study are included in this published article. Treatment data from 1990 to 2024 are available in the WHO repository [https://apps.who.int/iris/]. The dataset analyzed is available from the National Neglected Tropical Disease Program on reasonable request and can be made available with permission from the Ministry of Health and Sanitation, Sierra Leone.

## Abbreviations

CI: Confidence interval
IgG4: Immunoglobulin G4
LF: Lymphatic filariasis
MDA: Mass drug administration
OCP: Onchocerciasis Control Program in West Africa
OTS: Onchocerciasis Technical Subgroup
RDT: Rapid diagnostic test
TAS: Transmission assessment survey
WHO: World Health Organization
